# A Murine Model of X-Linked Moesin-Associated Immunodeficiency (X-MAID) Reveals Defects in T Cell Homeostasis and Migration

**DOI:** 10.3389/fimmu.2021.726406

**Published:** 2022-01-06

**Authors:** Lyndsay Avery, Tanner F. Robertson, Christine F. Wu, Nathan H. Roy, Samuel D. Chauvin, Eric Perkey, Ashley Vanderbeck, Ivan Maillard, Janis K. Burkhardt

**Affiliations:** ^1^ Department of Pathology and Laboratory Medicine, Children’s Hospital of Philadelphia Research Institute, Philadelphia, PA, United States; ^2^ Perelman School of Medicine, University of Pennsylvania, Philadelphia, PA, United States; ^3^ Graduate Program in Cellular and Molecular Biology and Medical Scientist Training Program, University of Michigan, Ann Arbor, MI, United States; ^4^ Division of Hematology/Oncology, Department of Medicine and Abramson Family Cancer Research Institute, Perelman School of Medicine, University of Pennsylvania, Philadelphia, PA, United States

**Keywords:** T cell, immunodeficiency, actin, moesin, migration, cytoskeleton, hematopoiesis, development

## Abstract

X-linked moesin associated immunodeficiency (X-MAID) is a primary immunodeficiency disease in which patients suffer from profound lymphopenia leading to recurrent infections. The disease is caused by a single point mutation leading to a R171W amino acid change in the protein moesin (moesin^R171W^). Moesin is a member of the ERM family of proteins, which reversibly link the cortical actin cytoskeleton to the plasma membrane. Here, we describe a novel mouse model with global expression of moesin^R171W^ that recapitulates multiple facets of patient disease, including severe lymphopenia. Further analysis reveals that these mice have diminished numbers of thymocytes and bone marrow precursors. X-MAID mice also exhibit systemic inflammation that is ameliorated by elimination of mature lymphocytes through breeding to a Rag1-deficient background. The few T cells in the periphery of X-MAID mice are highly activated and have mostly lost moesin^R171W^ expression. In contrast, single-positive (SP) thymocytes do not appear activated and retain high expression levels of moesin^R171W^. Analysis of *ex vivo* CD4 SP thymocytes reveals defects in chemotactic responses and reduced migration on integrin ligands. While chemokine signaling appears intact, CD4 SP thymocytes from X-MAID mice are unable to polarize and rearrange cytoskeletal elements. This mouse model will be a valuable tool for teasing apart the complexity of the immunodeficiency caused by moesin^R171W^, and will provide new insights into how the actin cortex regulates lymphocyte function.

## Introduction

Protective immune responses depend on regulated actin cytoskeletal dynamics, which direct cell migration, adhesion and signaling (1–5). The importance of these processes is highlighted by the existence of several primary immunodeficiency diseases linked to mutations in actin regulatory proteins like WASp, WIPF1, Rac2, Hem1, and Dock2 (6–8). Recently, a new primary immunodeficiency disorder was described and attributed to mutations in the actin binding protein moesin (9–12). Unlike other actin regulatory proteins linked to immunodeficiency, moesin does not regulate actin filament growth. Instead, it reversibly links the cortical actin cytoskeleton to plasma membrane lipids and proteins, thereby providing structural rigidity to the cell, controlling cell shape changes, and organizing specialized membrane domains (13, 14).

Moesin is a member of the ezrin/radixin/moesin (ERM) protein family. One or more members of this highly homologous group of proteins is expressed in most cell types; T cells express high levels of moesin, moderate levels of ezrin, and little to no radixin (15). These proteins are comprised of an N-terminal 4.1-ezrin-radixin-moesin (FERM) domain, a flexible linker, and a C-terminal actin binding domain (ABD). In the active conformation, the FERM domain of moesin associates with the plasma membrane by binding to phosphatidylinositol bisphosphate (PIP_2_) and to the cytoplasmic tails of membrane proteins such as CD43, while the ABD interacts with actin filaments that lie just beneath the membrane. Moesin can also assume an inactive conformation, in which intramolecular interaction of the FERM and ABD domains (16) masks the binding sites for plasma membrane components and actin. PIP_2_ binding and phosphorylation at T558 residue disrupt the autoinhibited fold, activating linker activity (17–21). Engagement of antigen or chemokine receptors leads to transient ERM protein dephosphorylation and loss of linker activity, allowing molecular rearrangements and cell shape changes associated with T cell activation and migration (22–24). When overexpressed in cells, constitutively active mutants of ezrin or moesin prevent appropriate cytoskeletal rearrangement. Lymphocytes expressing these mutants are abnormally rigid, polarize poorly, and display defective migratory responses *in vitro* and *in vivo* (22, 23, 25, 26). Interestingly, however, cells lacking ERM proteins or lymphocyte-oriented kinase (LOK), the kinase that activates linker activity, polarize well and migrate relatively normally in response to conventional chemokines (27, 28). Nonetheless, moesin knockout mice exhibit profound lymphopenia, due in large part to defective migratory responses to sphingosine-1-phosphate (29). Though T cells express both ezrin and moesin, deletion of ezrin in the T cell compartment has little effect on T cell trafficking (15), and the effect of deleting both ezrin and moesin is only slightly more severe than deleting moesin alone (29), indicating that moesin is the most important ERM family member in T cells.

Recently, a dozen patients worldwide have been diagnosed with a novel combined immunodeficiency disease known as X-linked moesin-associated immunodeficiency (X-MAID) (9–12). Remarkably, eleven of the twelve patients have the same single point mutation within the moesin FERM domain (R171W). Disease is characterized by severe lymphopenia, fluctuating neutropenia, and recurrent viral and bacterial infections. Most patients also exhibit eczema and other autoimmune phenotypes. Disease severity is variable; some patients required bone marrow transplantation although most patients have responded well to IVIG, prophylactic antibodies, and/or G-CSF (9, 11, 12). Little is known about the cell biological basis for pathology in X-MAID, though analysis of patient PBMCs shows evidence of defects in proliferation and migration (11). Furthermore, data from patient cells point to complex phenotypic changes related to patient age and lymphocyte activation status. In order to better understand this disease, we used CRISPR technology to generate mice with germline expression of moesin^R171W^ (note that murine and human moesin are 99% identical at the amino acid level, and 100% identical within the FERM domain where the mutation lies). This X-MAID mouse model recapitulates key aspects of the human disease including profound lymphopenia and susceptibility to opportunistic infections. X-MAID mice exhibit diminished numbers of thymocytes and bone marrow precursors, and systemic inflammation that can be ameliorated by mature lymphocyte deletion. The few peripheral T cells that are present are highly activated and have lost moesin expression, whereas SP thymocytes express high levels of the mutant protein. Functional analysis of X-MAID thymocytes reveals defects in migration traceable to an inability to undergo appropriate chemoattractant-induced cell shape changes. This mouse model provides novel insights into the mechanisms underlying moesin-based immunodeficiency.

## Materials and Methods

### Mice

Moesin knockout (MKO) mice on the C57BL/6 background were described previously (28–30). Mice homozygous for Rag1^tm1mom^ (RagKO) were obtained from Jackson Laboratories and bred in house. CRISPR mice in which the murine moesin gene was edited to contain the R171W mutation found in the majority of X-MAID patients (X-MAID mice) were generated by the CRISPR/Cas9 Mouse Targeting Core Facility, together with the Transgenic and Chimeric Mouse Facility at the University of Pennsylvania, following protocols published in (31). Briefly, Cas9 mRNA, gRNA, and ssDNA oligos containing the R171W X-MAID point mutation were designed and injected into C57BL/6J zygotes. Embryos were then transferred into pseudopregnant mice, creating the F0 chimeric generation. All founder mice were screened for chimerism and bred to determine germline transmission of the mutation. X-MAID mice were backcrossed to C57BL/6J mice (Jackson Laboratories) for at least 4 generations. Results from two independent founder lines were in agreement. All mice were bred in-house under SPF conditions and used at 3-5 weeks of age, all in accordance with protocols approved by the Institutional Animal Care and Use Committee of the Children’s Hospital of Philadelphia Research Institute. Exclusively male mice were used of every genotype, with WT mice being littermates to X-MAID mice.

### Histology

Upon necropsy, mice were perfused with 10% formalin *via* the pulmonary artery to inflate and fix the lung tissue. Tissues were collected into 10% formalin and processed by the Pathology Core Facility at the Children’s Hospital of Philadelphia. Briefly, tissue was paraffin embedded and then five-micron sections were cut and stained with hematoxylin and eosin. Slides were digitally scanned at 20× magnification on a Leica DM4000B upright imaging scope with a Spot RT/SE Slider Camera.

### Flow Cytometry

Single cell suspensions were prepared from spleens, thymi, and bone marrow while peripheral blood mononuclear cells were isolated with Lymphoprep (STEMCELL). For surface labeling, the following reagents from Tonbo Biosciences were used: Ghost Dye (Live/Dead) in v510, CD44 (IM7) in APC-Cy7, CD62L (MEL-14) in APC, TCRβ (H57-597) in PE or FITC, CD25 (PC61.5) in PE-Cy7, NK1.1 (PK136) in FITC, and Ly6G (1A8) in PerCP-Cy5.5. Antibodies to CD8 (53-6.7) in BV711, and CD4 (GK1.5) in BUV395 were from BD Biosciences, and antibodies to CD69 (H1.2F3) in APC, CD19 (6D5) in BV785, CD11b (M1/70) in AlexaFluor700, Ly6C (HK1.4) in Pacific Blue, CD29 (HMβ1-1) in FITC, LFA-1 (H155-78) in PE, Flt3 (A2F10) in APC, Ckit (ACK2) in APC-Cy7 or APC, CD150 (TC15-12F12.2) in PE-Cy7, CD48 (HM48-1) in FITC, IL7Rα (A7R34) in v450, CD41 (MWReg30) in APC-Cy7, FcγRII/III (93) in BV711, Sca1 (D7) in PerCP-Cy5.5, and CD105 (MJ7/18) in v450 were from Biolegend. In addition, the lineage dump gate included antibodies for CD3, CD8α, CD11b, CD11c, CD19, B220, TCRβ, TCRγδ, GR-1, NK1.1, and Ter119 all in PE conjugate format from Biolegend. For intracellular staining, surface staining was followed by fixation/permeabilization with the FoxP3 fix/perm kit (eBioscience), then cells were labeled for 30 minutes at room temperature with rabbit anti-moesin (Q480, Cell Signaling Technologies) followed by anti-rabbit AlexaFluor 647 secondary antibody (Invitrogen), and/or with anti-FoxP3 (FJK-16s) in PerCP-Cy5.5 from eBioscience. For flow cytometric analysis of F-actin polymerization, cells were stimulated with CCL19 (100 ng/ml) and fixed with 3% paraformaldehyde in PBS at the indicated times. Cells were then permeabilized with PSG (PBS, 0.01% saponin, 0.25% fish skin gelatin) and labeled with phalloidin AlexaFluor 488 (Invitrogen). All samples were analyzed on an LSRII (BD Biosciences) equipped with FACSDiva software (BD Biosciences). Data were analyzed using FlowJo software (v.10.4.2).

### Western Blotting

CD4 SP thymocytes from WT and X-MAID mice were isolated by negative selection using a magnetic CD4^+^ T cell isolation kit (Miltenyi). Cells were starved for 4 hours in serum-free DMEM (Corning) before stimulation 100ng/ml CCL19 (R&D Systems) for the indicated times. Cells were then lysed with 1% Triton X100, 50 mM Tris-HCl, 50 mM NaCl, 5 mM EDTA, 50 mM NaF, 30 mM Na_4_P_2_O_7_, 50 mM β-glycerophosphate, with Roche Complete protease inhibitors. Proteins were separated by SDS/PAGE (4-12% NuPAGE bis-tris gel, Invitrogen) transferred to nitrocellulose membranes, blocked with Odyssey buffer (Licor) and probed for the indicated proteins. Antibodies to pERK1/2 (Thr202/Tyr204) and pAkt (Ser473) were from Cell Signaling Technologies. GAPDH was used as a loading control, and was detected with Clone 6C5 antibody, from Sigma. Primary antibodies were detected with fluorescent secondary antibodies (anti-rabbit AlexaFluor 800 or anti-mouse AlexaFluor 680, from Invitrogen). Blots were imaged using an Odyssey fluorescence-based imaging system (Licor) and prepared for publication using ImageLite Software (Licor).

### Transwell Assays

CD4 SP thymocytes from WT and X-MAID mice were isolated by negative selection using a magnetic CD4^+^ T cell isolation kit (Miltenyi) and resuspended in DMEM with 10% charcoal stripped FBS (Gibco). 2x10^5^ cells per well were placed in the top chambers of a 24 well transwell plate (5μm pore size, Corning) and allowed to settle at 37°C for 10 minutes. The top chambers were then placed on top of bottom chambers containing 100ng/ml CCL19, and incubated for 2 hours at 37°C. Top chambers were then removed and cells in the bottom chambers were counted using a hemocytometer. Percent migration was calculated based on input cell numbers.

### Microscopy

Live cell imaging was conducted essentially as described previously (32). Eight-well chamber slides (Lab-Tek) were coated with 2μg/ml of murine ICAM-1-Fc (R&D Systems). CD4 SP thymocytes were placed in Leibovitz’s L-15 media (Gibco) supplemented with 2mg/ml glucose and 0.1% FBS and incubated for 20 minutes. Cells were then added to ICAM-1 coated chambers, allowed to settle for 10 minutes, then imaged using a DIC 10× lens on a Zeiss Axiovert 200M microscope and ORCA-ER CCD camera (Hamamatsu). Time-lapse images were collected every 30s for 10 minutes using Slidebook 6 software (Intelligent Imaging Innovations). Migration was quantified using the manual tracking plugin in ImageJ. Cells that were not alive, not attached to the glass, or left the frame during the time frame were excluded from analysis. For each cell that met the criteria for inclusion, the total distance traversed was determined, as was the net displacement (defined as the distance between the final and starting coordinates of the cell). The percentage of migrating cells was calculated based on counting cells with a net displacement of at least 10 microns, divided by the total number of live cells that were attached to the cover glass.

Cell polarization was assessed based on immunofluorescence microscopy of fixed cells. CD4 SP thymocytes were stimulated in suspension with 100ng/ml CCL19 for indicated times, after which they were fixed with 4% paraformaldehyde in PBS and attached to coverslips coated with poly-L lysine. Cells were then permeabilized using PSG and stained with phalloidin AlexaFluor 488 and rat anti-tubulin (clone YL1/2, Millipore/Sigma) followed by goat anti-rat AlexaFluor 647 secondary antibody (Invitrogen). Slides were mounted in Mowiol and images were collected on an Axiovert 200M (Zeiss) with a spinning disk confocal system (UltraVIEW ERS6; Perkin Elmer) equipped with an ORCA-Flash 4.0 CMOS camera (Hamamatsu) and a 63× 1.4 NA Planapo objective. Images were acquired using Volocity v6.3 software (Perkin Elmer) and were prepared for publication using ImageJ. Roundness was defined by first outlining cells in ImageJ using the wand tool, then choosing the shape descriptors option. The software determined cell roundness by the following formula: $4\times \frac{\left[Area\right]}{\pi \times {\left[Major\;axis\right]}^{2}}$ .

### mRNA Quantification

Single cell suspensions were made from spleens of WT, X-MAID, or MKO male mice. 1 WT, 1 MKO, and 4-5 X-MAID spleens were used for each experiment. Cells were enriched by negative selection using rat anti-CD8 hybridoma supernatant (Clone 53-6.7) and anti-MHC II (BioXCell) antibodies followed by anti-rat magnetic beads (Qiagen). CD4^+^ T cells were further purified by flow sorting using CD4-APC (Biolegend) with the FACSJazz (BD). RNA isolation was completed using the PureLink RNA mini kit (Invitrogen). Equal amounts of RNA were reverse transcribed from each group using the High Capacity RT-PCR kit (Applied Biosystems). The resulting cDNA was used for qPCR using *Msn*, *Ezr*, and *Gapdh* PrimeTime qPCR primers (IDT) and SYBR Green mastermix (Applied Biosystems). qPCR Ct was quantified on the 7500 Standard (Applied Biosystems). Data are represented as ΔCT^-1^ defined as the inverse of Ct of the primer of interest minus the *Gapdh* Ct for that sample. Technical duplicates were run for each sample.

### Statistical Analysis

Unless specified otherwise, data were analyzed using a 2-sided Student’s *t* test with alpha = 0.05. All analysis was conducted using GraphPad Prism Software, v. 9.1.2.

## Results

### X-MAID Mice Exhibit Opportunistic Infections and Lymphocyte-Dependent Inflammation

**Figure 1:**
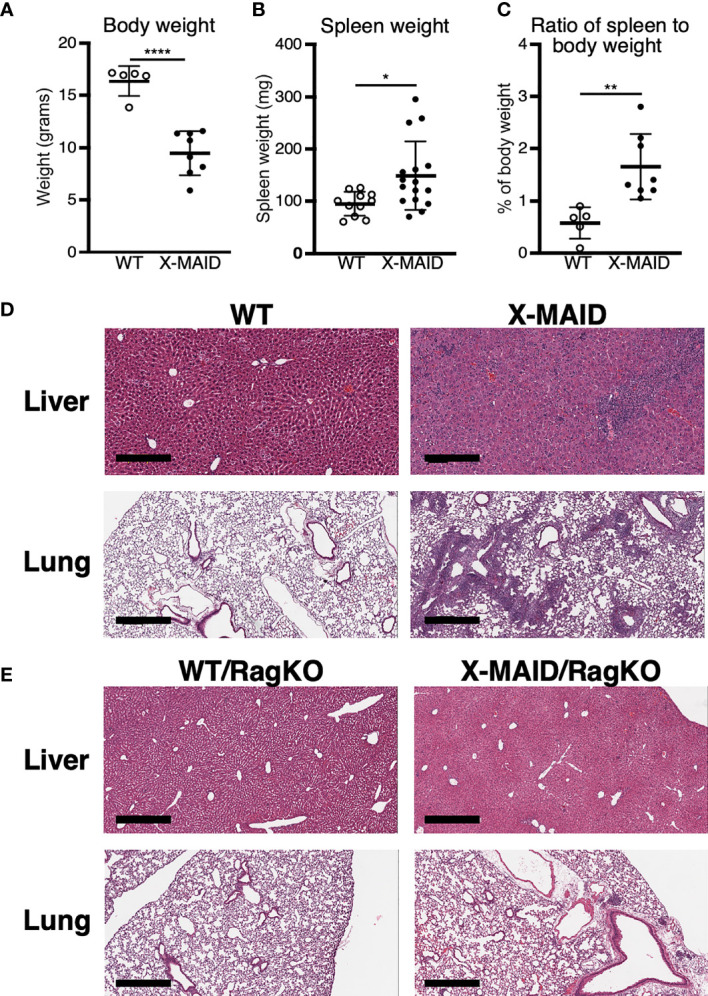
X-MAID mice exhibit systemic inflammation ameliorated by lymphocyte deletion. WT or X-MAID male mice at 3-4 weeks old were measured for **(A)** body weight and **(B)** spleen weight. **(C)** The ratio of spleen to body weight was calculated. **(D, E)** H&E stains of sections from liver and lung of the indicated mouse strains, all at 3-4 weeks of age. Scale bars = 600μm. Note the granulocytic infiltration in X-MAID tissues, which is absent in tissues from X-MAID/RagKO mice. Data in **(A–C)** represent means ± StDev, with each point representing an individual mouse. Statistics were calculated using a Student’s *t* test, *p < 0.05, **p < 0.01, ****p < 0.0001.

A novel combined immunodeficiency disease termed X-MAID has recently been described in patients bearing a specific point mutation in the actin linker protein moesin (9–12). To better understand the mechanistic basis of this disease, we generated CRISPR knock-in mice bearing the causative mutation (R171W, hereafter called X-MAID mice). Both male X-MAID mice and heterozygous females were born at normal Mendelian sex-linked ratios. Female heterozygotes were normal and fertile, although an increased rate of dystocia was noted. In contrast, hemizygous males exhibited partial (approximately 30%) perinatal lethality. The surviving mutant males developed numerous opportunistic infections in the skin, eye, and nasal cavity, as well as inflammatory infiltrates in multiple tissues, succumbing by 7-10 weeks of age. In order to minimize secondary effects of infection, we focused further analysis on young mice (3-5 weeks of age), before mice exhibit signs of infection. At this early age, X-MAID mice were runted and spleens were enlarged (**Figures 1A–C**). The elevated spleen-to-body-weight ratio observed in X-MAID mice is consistent with systemic inflammation. In addition, histological analysis of the lung and liver showed granulocytic infiltration and evidence of extra-medullary hematopoiesis (**Figure 1D**).

To ask if the inflammatory phenotype observed in X-MAID mice is attributable to primary defects in the lymphoid compartment, X-MAID mice were bred to Rag1^-/-^ (RagKO) mice, to generate X-MAID mice lacking mature lymphocytes. As shown in **Figure 1E**, this ameliorates the inflammation of non-lymphoid tissues, indicating that lymphocytes are key drivers of pathology in X-MAID. In addition, X-MAID/RagKO mice survived as long as their RagKO counterparts (data not shown). We therefore focused our analysis on the lymphoid compartment, giving special attention to T cells, where defects in X-MAID patients are observed (9–12).

### Peripheral T Cell Numbers Are Reduced and Remaining Cells Are Highly Activated

**Figure 2:**
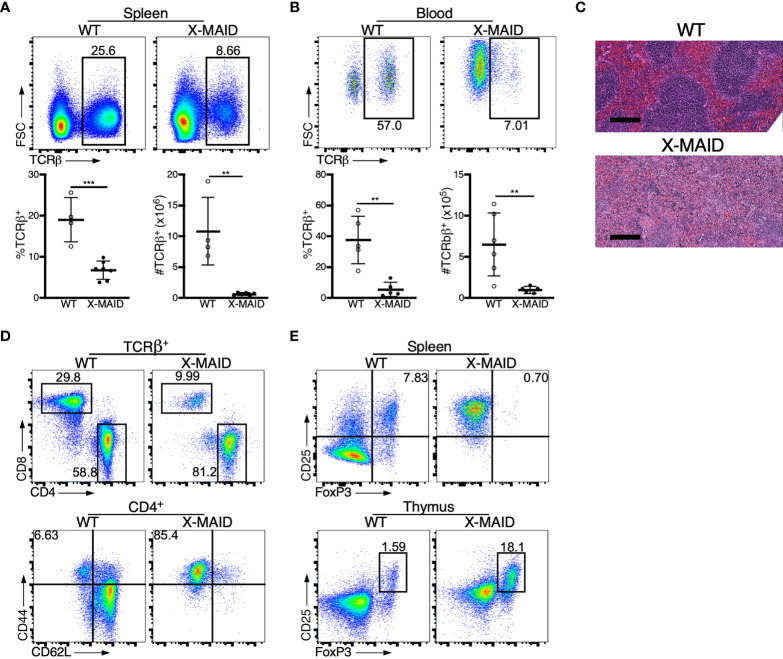
Dysregulation of T cell populations in peripheral lymphoid organs of X-MAID mice. WT or X-MAID male mice were sacrificed at 3-5 weeks old, and the indicated tissues were harvested for analysis. **(A, B)** Using flow cytometry, gated on live single cells, the proportion and absolute number of TCR β^+^ cells were determined from **(A)** spleen or **(B)** blood. **(C)** H&E staining from spleen sections shows a loss of follicle structure. Scale bars = 200μm. **(D)** Representative flow plots of CD4^+^ and CD8^+^ T cell subsets gated on TCR β^+^ live single cells from the spleens of WT or X-MAID mice (top). Further analysis of CD44 and CD62L expression on CD4^+^ T cells reveals elevated activation status (bottom). **(E)** CD25^+^FoxP3^+^ cells from the spleen (top) or thymus (bottom) of WT or X-MAID male mice gated on CD4 SP live single cells. Data in C-E are representative of results from at least 6 individual mice. Data in **(A, B)** represent means ± StDev, with each point representing an individual mouse. Statistics were calculated using a Student’s *t* test, **p < 0.01, ***p < 0.005.

X-MAID patients exhibit reduced numbers of blood T and B cells (9–12). Consistent with this, X-MAID mice showed profound lymphopenia in both spleen and blood, and lymph nodes were nearly undetectable (**Figures 2A, B**, **S1**, and data not shown). Proportions of other immune cell types (NK cells, monocytes, and neutrophils) were elevated in the spleen, although this effect was largely due to the loss of lymphocytes; the absolute numbers of these cell types were either normal or modestly reduced in X-MAID mice (**Figure S1A** and data not shown). Similar results were obtained for these populations in the blood, except that circulating neutrophil numbers were clearly elevated in X-MAID mice (**Figures S1B, C**). Histological analysis revealed a lack of splenic architecture (**Figure 2C**). Further analysis of splenic T cells from X-MAID mice reveals increased CD4:CD8 ratios and a highly activated (CD44^+^CD62L^–^) phenotype for both CD4^+^ and CD8^+^ T cells (**Figures 2D**, **S2**). This activated phenotype, which is also observed in X-MAID patients, may be due to homeostatic proliferation induced by lymphopenia. However, regulatory T cells (T_regs_) were nearly undetectable in X-MAID spleens (**Figure 2E**), and this may also contribute to effector T cell activation. Note that although moesin has been implicated in iT_reg_ development (33), the lack of peripheral T_regs_ in X-MAID mice is not attributable to an inability to produce nT_regs_, as thymic nT_regs_ were readily detectable (**Figure 2E**).

### Thymocyte Numbers Are Low, but Development and Activation State Are Grossly Normal

**Figure 3:**
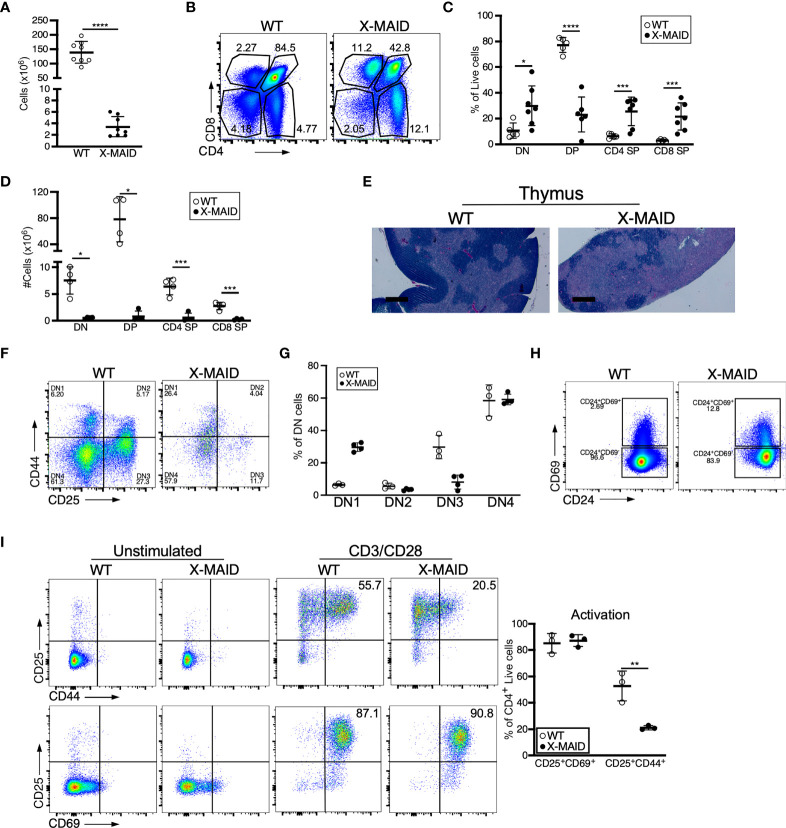
Thymocyte numbers are reduced in X-MAID mice, but development and activation are grossly normal. Thymi from 3-4 week-old WT or X-MAID male mice were harvested and single cell suspensions were generated. **(A)** Total thymocyte number **(B)** Representative flow plots of thymocyte populations, gated on live single cells. Proportions **(C)** and absolute numbers **(D)** of thymocyte subsets were quantified, with each point representing an individual mouse. **(E)** H&E staining of the thymi from WT and X-MAID mice (representative of 4 mice for each genotype). Scale bars = 400μm. **(F)** Representative flow plots of thymocytes prepared as in A-C stained with CD44 and CD25 and gated on the CD4^–^CD8^–^ (DN) live population. **(G)** The proportion of each DN population (DN1-DN4) is quantified with each point representing an individual mouse. **(H)** Representative flow plots of thymocytes prepared as in A-C stained with CD69 and CD24 gated on CD4^+^CD8^+^ live population. **(I)** CD4 SP Thymocytes from WT or X-MAID male mice were left unstimulated or stimulated overnight with CD3/CD28 and stained for CD25, CD69, and CD44. Representative plots are gated on CD4 SP live single cells (left) and the proportion of activated cells was quantified (right). Representative of 3 independent experiments. Data in **(A, C, D, G, I)** represent means ± StDev. Statistics were calculated using a Student’s *t* test, * p< 0.05, **p < 0.01, ***p < 0.005, ****p < 0.001.

Closer examination of thymi from X-MAID mice at weaning age showed significantly reduced cellularity as compared with WT littermates (**Figure 3A**). Proportions of DN and SP populations were elevated in thymi from X-MAID mice, while the proportion of DP cells was reduced (**Figures 3B, C**). However, because of the decreased cellularity, the absolute number of all thymic populations was reduced in X-MAID mice (**Figure 3D**). Histological analysis revealed reduced cortical area, consistent with the loss of DP cells (**Figure 3E**). Further flow cytometric analysis of DN populations based on CD44 and CD25 expression showed increased proportions of the DN1 population and decreased proportions of the DN3 population (**Figures 3F, G**). Notably, however, there was no significant accumulation of DN4 cells. Furthermore, analysis of DP thymocytes revealed an elevation in the proportion of CD24^+^/CD69^+^ population in X-MAID (**Figure 3H**), consistent with intact activation of these cells. Taken together, these findings suggest that the X-MAID mutation does not lead to gross defects in positive selection. Additional analysis will be required to definitively test this point and to determine any effects on the thymic repertoire.

Since peripheral CD4^+^ T cells from X-MAID mice exhibit high basal expression of activation markers, we asked if this process begins in the thymus. CD25 and CD69 expression was analyzed in freshly isolated CD4 SP thymocytes without stimulation, and after stimulation for 24h with anti-CD3 and anti-CD28. As shown in **Figure 3I**, baseline levels of surface CD25 and CD69 were normal. After stimulation, upregulation of these activation markers was similar to that in WT thymocytes, both in terms of the number of positive cells and the levels of surface marker expression. X-MAID thymocytes did exhibit diminished ability to upregulate the late-activation marker CD44; this appeared to be largely due to reduced surface expression levels. Since CD44 is known to interact directly with moesin (34), this may reflect an effect on CD44 trafficking rather than an impact on T cell activation status.

### X-MAID Mice Show Defects in Bone Marrow Precursor Populations

**Figure 4:**
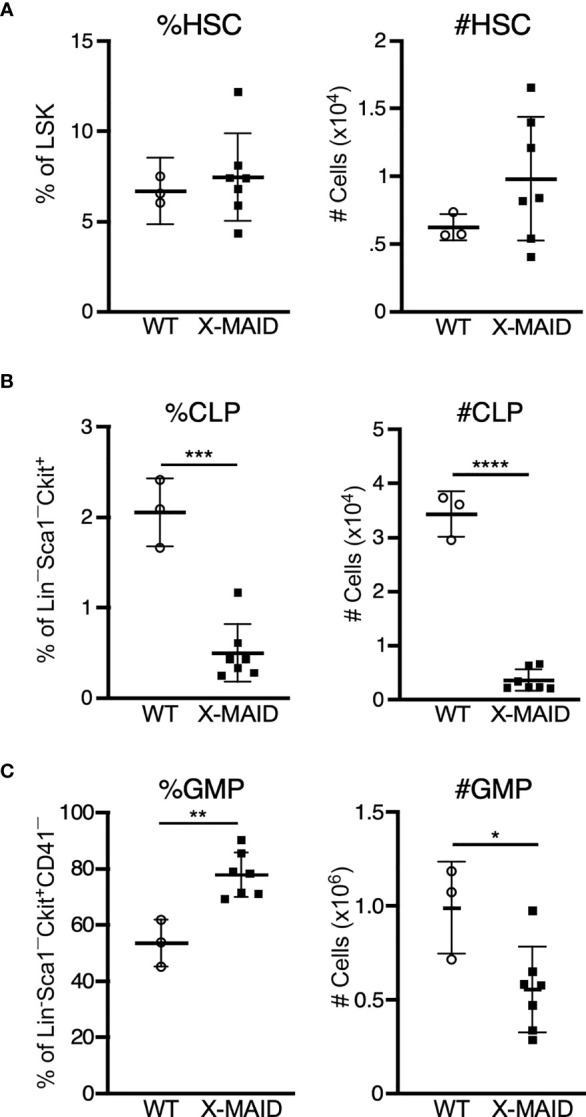
Bone marrow of X-MAID mice shows a depleted lymphocyte progenitor population. Bone marrow cells from 3-5 week-old WT or X-MAID male mice were harvested, counted, and processed for flow cytometry. **(A)** Proportion and absolute number of hematopoietic stem cells (HSC) (CD150^+^CD48^–^) displayed as gated on Lin^–^Sca1^+^Ckit^+^ (LSK) population. **(B)** Proportion and absolute number of common lymphoid progenitors (CLP) (Flt3^+^IL7Rα^+^) displayed are gated on Lin^–^Sca1^LOW^Ckit^LOW^. **(C)** Proportion and absolute number of granulocyte/macrophage progenitors (GMP) (%FcγRII/III^+^CD150^–^) displayed are gated on Lin^–^Sca1^–^Ckit^+^CD41^–^. Data represent means ± StDev, with each point representing an individual mouse. Statistics were calculated using a Student’s *t* test, *p < 0.05, **p < 0.01, ***p < 0.005, ****p < 0.001.

One of the most prominent features of X-MAID mice is overall paucity of thymocytes. To explore the basis of this defect, we conducted a series of pilot experiments using different bone marrow chimera models. Regardless of the experimental conditions, we found very few thymocytes in mice receiving X-MAID bone marrow (data not shown). This observation led us to examine bone marrow populations in X-MAID donors. Interestingly, we found that although the HSC population (as defined in (35), see also **Figure S3A**) appears relatively normal in X-MAID mice (**Figure 4A**), the absolute numbers and proportion of common lymphoid progenitors (CLPs) were severely reduced (**Figure 4B**). In contrast, we observed an increased proportion of granulocyte/macrophage progenitors (GMPs), although the absolute numbers of these cells were slightly lower than normal (**Figure 4C**). Based on these findings, we examined the lymphoid-primed multipotent progenitor (LMPP) population, which gives rise to CLPs and found significantly fewer cells in this population (**Figure S3D**). However, the multipotent progenitor (MPP) population, which gives rise to LMPPs as well as GMPs, was intact (**Figure S3C**) (36). Therefore, defects in cell numbers were first evident at the LMPP stage. Notably, there are also significantly fewer megakaryocyte precursors (MkP), but not erythroid progenitors [EryP (37)] in X-MAID bone marrow (**Figures S3E, F**). At present, almost nothing is known about the expression patterns and functional role of moesin with bone marrow precursor populations. Thus, additional work will be needed to understand why the moesin^R171W^ mutant protein is deleterious for LMPP cells, and whether this effect is cell-autonomous or indirect. Nonetheless, since LMPPs and CLPs are the primary precursor populations that exit the bone marrow and settle in the thymus, it seems likely that the low number of these precursor cells is at least partially responsible for the dearth of thymocytes in X-MAID mice.

### Moesin^R171W^ Is Highly Expressed in the Thymus and Lost in the Periphery

**Figure 5:**
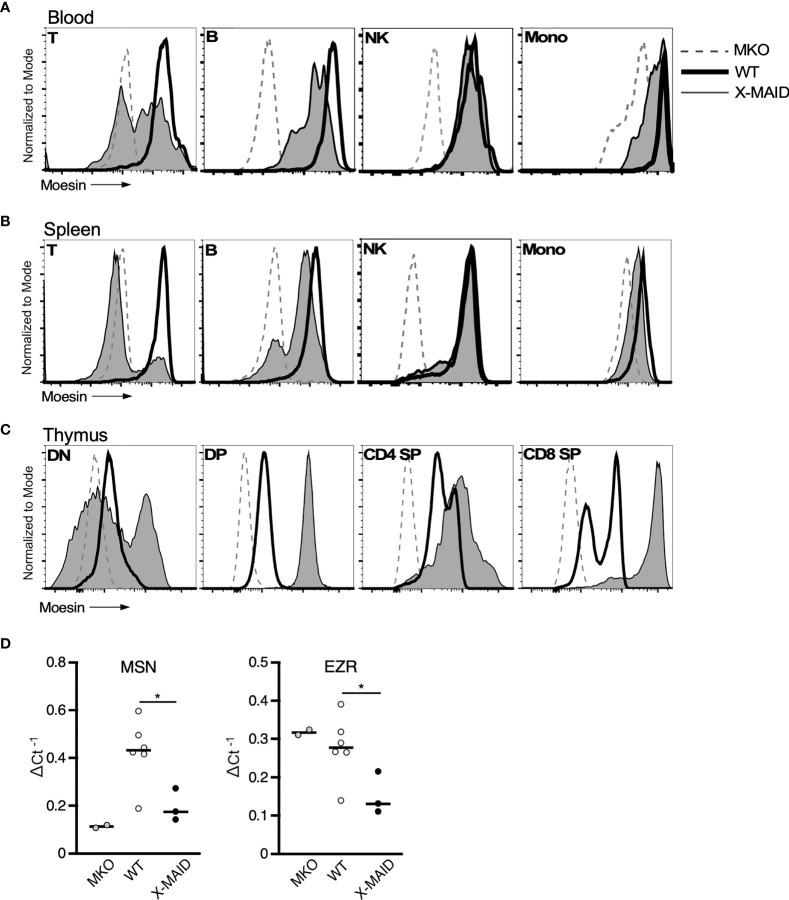
Moesin^R171W^ expression is selectively lost in peripheral T cells. 3-4 week-old WT, X-MAID, or MKO male mice were sacrificed and the indicated tissues were harvested and processed for single cell suspensions. Cells were labeled for surface markers, and for intracellular moesin. For blood **(A)** and spleen **(B)**, populations were gated on live single cells, followed by TCRβ^+^ (T), CD19^+^ (B), NK1.1^+^ (NK), and CD11b^+^Ly6C^+^ (Mono). For thymus **(C)**, populations were gated on live single cells, followed by CD4^–^CD8^–^ (DN), CD4^+^CD8^+^ (DP), CD4 SP, or CD8 SP. **(D)** Relative *Msn* (left) and *Ezr* (right) mRNA expression in CD4^+^ splenic T cells from WT, X-MAID, or MKO male mice. Expressed as the inverse of the Ct of indicated gene subtracted from that of *Gapdh*. Bars in D represent means. Statistics between WT and X-MAID groups were calculated using a Student’s *t* test, *p < 0.05.

The immunodeficiency and inflammatory phenotypes that we observe in X-MAID mice differ dramatically from subtle abnormalities seen in mice bearing a germline deletion of moesin (MKO mice) (28–30). This suggests that in X-MAID, expression of the mutant moesin protein is important for disease. In keeping with this view, in X-MAID patients, the mutant protein is expressed in some peripheral T cells, but is selectively lost with time and/or activation (9, 11). To ask if expression and loss of the mutant protein is recapitulated in the X-MAID mouse model, we compared the expression levels of moesin in peripheral leukocytes from X-MAID, MKO and WT mice. Analysis of blood T cells from X-MAID mice revealed a pattern of protein loss similar to that described in humans (**Figure 5A**). Moesin expression was also reduced in CD19^+^ B cells but this effect was much more modest than in T cells. Analysis of splenic T and B cells revealed more profound loss of expression; most splenic T cells and a subset of B cells had extinguished expression altogether (**Figure 5B**). Loss of moesin expression was specific to B and T cells; moesin^R171W^ expression was retained at WT levels in other immune populations, including natural killer cells, monocytes, and granulocytes (**Figures 5A, B**, and data not shown). Interestingly, although expression of moesin^R171W^ is silenced in peripheral lymphocytes, expression levels are elevated in thymic populations (**Figure 5C**). This is particularly clear starting at the DP stage, a time when we have previously shown that moesin expression is upregulated (15).

To better understand the striking downregulation of moesin expression that takes place in peripheral T cells, we asked if moesin mRNA levels are affected by conducting qPCR analysis on splenic CD4^+^ T cells from WT, MKO, and X-MAID mice. As shown in **Figure 5D**, moesin mRNA levels were significantly reduced in T cells from X-MAID mice. Indeed, the levels were just above the baseline set by MKO cells. Unexpectedly, we found that ezrin mRNA levels were also significantly reduced in X-MAID T cells. This contrasts with MKO T cells, where ezrin mRNA is expressed at WT levels. Taken together, these data show that the moesin^R171W^ gene encodes a protein that is expressed in leukocytes but is selectively downregulated in peripheral lymphocytes, and that downregulation occurs at the mRNA level. The fact that downregulation depends on cell type and developmental stage makes it unlikely that the moesin mutation directly impairs transcriptional efficiency or message stability. Instead, it seems possible that expression of the mutant protein has deleterious effects on cell function, which pressures cells to downregulate expression of the mutant protein. Since the ezrin locus is also affected, this feedback mechanism may involve common transcriptional regulatory factors.

### X-MAID T Cells Have Broad Migration Defects Due to the Inability to Polarize

**Figure 6:**
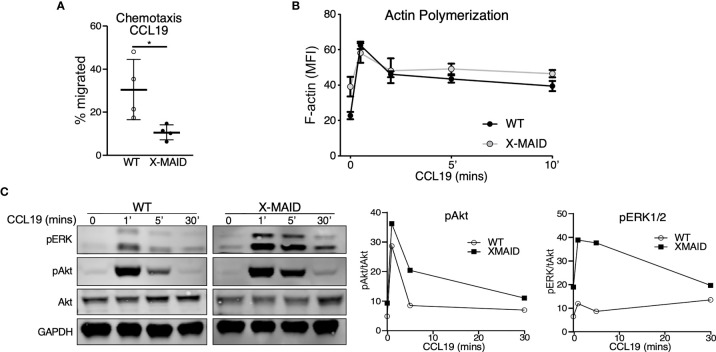
X-MAID thymocytes are unable to properly chemotax despite intact signaling. WT or X-MAID CD4 SP thymocytes were **(A)** placed in the upper chamber of a 5 μm pore transwell with 100ng/ml CCL19 in the media of the bottom chamber and incubated at 37 degrees for 2 hours. Cells in the bottom chamber were counted and percent migrated calculated. Graph shows results from 4 independent experiments, with each data point representing an average of three technical replicates from one experiment. Each experiment used pooled cells from 3-5 X-MAID mice, and cells from one matched WT littermate control. Data represent means ± StDev. Statistics were calculated using a Student’s *t* test, *p < 0.05. **(B)** WT or X-MAID CD4 SP thymocytes were left unstimulated, or stimulated with 100ng/ml CCL19 for the indicated times, permeabilized and stained with phalloidin to assess polymerized F-actin by flow cytometry. **(C)** Thymocytes stimulated as in B were fixed, lysed, and immunoblotted for pAkt (Ser473), pERK (Thr202/204), and total Akt and GAPDH as loading controls (left). WT and X-MAID samples were handled and analyzed in parallel. Blots were quantified by densitometry, and relative values were calculated after normalization to total Akt (right). For **(B, C)**, each experiment used pooled cells from 3-5 X-MAID mice, and cells from one matched littermate control. Results are representative of 3 independent experiments.

The selective loss of moesin^R171W^ in mature lymphocytes suggests that expression of the mutant protein is particularly deleterious for these cells. One known function of moesin in T cells is as an organizer of cell migration. Indeed, either deletion of moesin or expression of constitutively active ERM proteins causes defects in lymphocyte migration *in vitro* and *in vivo* (22, 25, 26, 28). We therefore asked if expression of the X-MAID point mutant disrupts cell migration. Because CD4 SP thymocytes are the most mature population that still express the mutant protein in most cells, we used this population for analysis. We first asked if CD4 SP thymocytes could migrate toward the chemoattractant CCL19 using a transwell assay system. As shown in **Figure 6A**, significantly fewer X-MAID thymocytes showed chemotactic responses in this assay. The failure of X-MAID thymocytes to chemotax efficiently does not reflect an inability of these cells to sense chemokine or signal through the receptor, because stimulation with CCL19 induced actin polymerization as well as phosphorylation of ERK and Akt in mutant thymocytes (**Figures 6B, C**). Indeed, CCL19-induced ERK phosphorylation was consistently elevated in X-MAID thymocytes. The reason for this remains unclear, though it may reflect dysregulation of inositol lipid homeostasis and Ras signaling responses.

**Figure 7:**
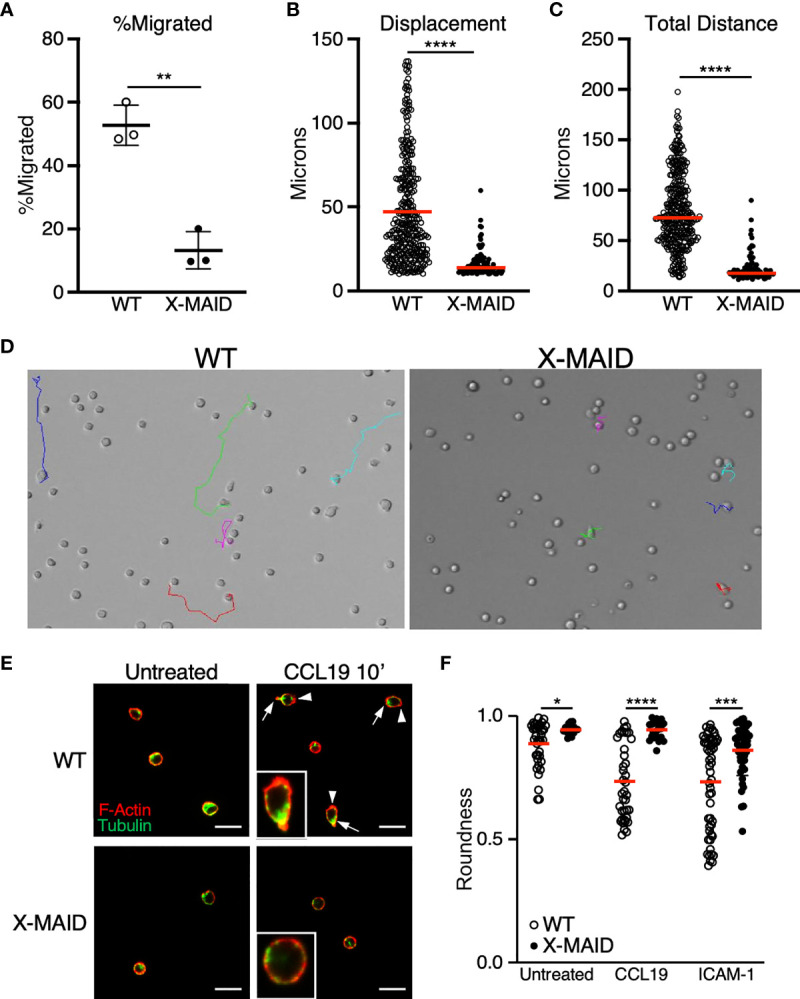
Reduced migratory capability is associated with lack of polarization in X-MAID T cells. **(A–D)** WT or X-MAID CD4 SP thymocytes were placed on ICAM-1 coated glass surfaces and imaged live using DIC optics every 30 seconds for 10 minutes. Cells were tracked using the manual tracking plugin on ImageJ. **(A)** % migrated was calculated as any cell moving more than 10μm and remaining in view for the entirety of the movie. Each point represents a field of at least 100 cells. Data represent means ± StDev. **(B)** Displacement and **(C)** Total distance traveled were calculated from first frame to final frame. **(D)** Representative images of the final frame with colored tracks from individual cells. **(E)** CD4 SP thymocytes were left untreated or stimulated with 100ng/ml CCL19 for 10 minutes, then fixed to a poly-L coated surface and stained for F-actin and tubulin to visualize actin leading edge (arrowheads) and tubulin-rich uropods (arrows). Representative images are shown for each condition, insets show higher magnification views of typical cells. Scale bars = 20μm **(F)** Images like those in **(D, E)** were used to measure roundness of cells using ImageJ software as detailed in the Materials and Methods. Means ± StDev are shown. **(A–D)** show data from one experiment (performed using cells pooled from 3-5 mice). This experiment was done twice with separate pools of mice, with similar outcomes. The data in **(E, F)** are pooled from two separate experiments done on different days, with each experiment using cells pooled from 3-5 mice. Data from the two experiments were in agreement, so were pooled for presentation. In **(A)**, each point represents one field of cells; In **(B, C, F)** each point represents a single cell. Red bars in **(B, C, F)** represent means. Statistics for all panels were calculated using a Student’s *t* test, *p < 0.05, **p < 0.01, ***p < 0.005, ****p < 0.001.

We next used live cell imaging to better define the migratory phenotype of X-MAID thymocytes. Since chemokine signaling is intact, we hypothesized that these cells exhibit general defects in motility. To test this, we imaged cells undergoing random migration on surfaces coated with integrin ligands. CD4 SP thymocytes from WT and X-MAID mice were observed while migrating on the LFA-1 ligand, ICAM-1, using live cell microscopy. In agreement with the transwell data, we found that whereas about 50% of WT thymocytes migrated in this assay (defined as having a net displacement of at least 10μm), only about 10% of X-MAID thymocytes met this criterion (**Figure 7A**). Moreover, even the motile subset of X-MAID thymocytes traveled a much shorter distance than WT cells, as measured by net displacement and total track length (**Figures 7B, C**). By observing the tracks of individual cells, the difference in migratory behavior between WT and X-MAID thymocytes is readily observed. Whereas a subset of WT thymocytes traveled significant distances along relatively linear paths, the motile X-MAID thymocytes showed only wobbling movement around their starting positions (**Figure 7D** and **Supplemental Movies 1**, **2**). We confirmed that this reduction in migration on ICAM-1 is not due to differences in integrin expression (**Figure S4**).

In order to migrate properly on integrin ligands, T cells must first polarize to form an elongated shape, with an actin-rich leading edge and a trailing, tubulin-rich uropod. In the course of analyzing our DIC movies, we frequently observed this type of polarized morphology in the WT thymocyte population, whereas the X-MAID cells seemed to maintain a rounded shape. To ask if X-MAID cells fail to elongate and undergo proper polarization of cytoskeletal elements, CD4 SP thymocytes from WT or X-MAID mice were left untreated or treated in solution with CCL19, fixed, and labeled with phalloidin and anti-tubulin antibodies. Cells were then observed by fluorescence microscopy and polarity was quantified as detailed in Materials and Methods. As shown in **Figure 7E**, unstimulated WT and X-MAID thymocytes were mostly quite round, with F-actin distributed over of the circumference of the cell, and the microtubule organizing center (MTOC) located at a random spot. After exposure to CCL19, a high proportion of WT cells become polarized, forming a clear actin-rich leading edge (arrowheads) and an opposing uropod marked by the MTOC (arrows). This segregation of cytoskeletal elements was accompanied by overall cell elongation, measured as a decrease in roundness (**Figure 7F**). In contrast, CCL19 stimulated X-MAID thymocytes looked very similar to unstimulated controls, with no clear rearrangement of actin and tubulin, and no reduction in roundness. A similar defect was observed in X-MAID thymocytes undergoing polarization in response to integrin engagement in the absence of chemokine (**Figure 7F**). While X-MAID thymocytes did show some elongation under these conditions, the response was blunted significantly. Taken together, these findings demonstrate that X-MAID thymocytes are unable to undergo appropriate morphological rearrangements necessary for motility, resulting in a general defect in cell migration.

## Discussion

Here, we describe a new mouse model that faithfully reflects many key features of human X-MAID immunodeficiency disease. Like their human counterparts, X-MAID mice exhibit profound lymphopenia and suffer from multiple opportunistic infections. The few T cells that are present in the periphery have a highly activated phenotype, and most have lost moesin expression. It seems likely that the extensive lymphocyte activation that we observe arises due to homeostatic proliferation occurring in response to severe lymphopenia, together with the profound loss of peripheral Tregs. Along with defects in lymphocytes, X-MAID patients also typically exhibit fluctuating neutropenia (9–12). We did not observe this in our mouse model. In fact, we observed an increased proportion of neutrophils in the blood and spleen at 4-5 weeks of age (**Figure S1C**). It remains unclear whether the apparent lack of neutropenia reflects a real difference between mice and humans, or if it is due to other factors, such as the lack of genetic variability in laboratory mice, housing in an SPF-facility, or timing of analysis (we did not analyze neutrophil numbers in a single mouse over time). Additionally, the opportunistic infections can induce neutrophilia that may not be seen in patients receiving prophylactic antibiotics.

Using the X-MAID mouse model, we could examine tissues that are inaccessible in the patients. Analysis of peripheral lymphoid organs revealed a loss of tissue architecture in the spleen, and an almost complete absence of lymph nodes. In the thymus, all developmental populations were present, suggesting that progression through thymic development occurs relatively normally. We did note a significant increase in the proportion SP thymocytes, possibly reflecting a defect in egress similar to that observed in moesin-deficient mice (29). Importantly, however, absolute numbers of thymocytes were severely reduced and thymic architecture was perturbed. Although Lagresle-Peyrou et al. (11) reported that X-MAID patients appear to have normal sized thymi, the same study and several others show that X-MAID patients have abnormally low T cell receptor excision circles (TRECs) (9–11), an indicator of low thymic output (38).

Analysis of bone marrow populations in X-MAID mice revealed a decrease in progenitor populations that are responsible for seeding the thymus. Since little is known about ERM protein expression or function within the bone marrow compartment, it is unclear why specific precursor populations are selectively impacted by expression of the mutant protein. One possibility is that the mutant protein is expressed in these cells and is deleterious for their proliferation or survival. However, since systemic stress suppresses LMPPs and CLPs (39), the reduction in these lineages could be a secondary effect of systemic stress due to infection, uncontrolled autoimmune inflammation, or malnutrition. A full understanding of how the X-MAID mutant protein impacts hematopoietic development will require the generation of an inducible mouse model, but our results indicate that the peripheral lymphopenia observed in X-MAID patients probably reflects defects in both bone marrow and thymic populations.

Another area where analysis of the X-MAID mice has proven to be illuminating concerns the regulation of protein levels. In X-MAID patients, moesin expression is lost in peripheral T and B cells, and this loss increases with patient age and cellular activation status (9, 11). Consistent with this, we observed loss of expression in peripheral lymphocytes of X-MAID mice, along with elevation of activation markers. In the mouse model, we could also analyze moesin expression in thymic populations, thereby obtaining information that is unavailable from X-MAID patients. Interestingly, we found that thymocytes express the mutant protein efficiently. Indeed, expression of the mutant protein in X-MAID thymocytes is several-fold higher than in the corresponding WT populations. QPCR analysis showed similar moesin mRNA levels in WT and X-MAID thymocytes (data not shown), so overexpression likely occurs at the protein level. We are currently testing the possibility that the mutant protein is at least partially misfolded, and that its turnover is slowed.

Unlike upregulation of moesin in the thymus, we find that downregulation in peripheral T cells occurs at the mRNA level. This finding is consistent with observations in peripheral T cells from X-MAID patients, which showed diminished moesin mRNA levels (9, 11). Since this loss is dependent on cell type and differentiation state, it seems likely that mRNA downregulation involves feedback inhibition rather than direct effects of the moesin mutation on transcriptional rates or message stability. This is further supported by our finding of diminished ezrin mRNA levels in X-MAID T cells. While ezrin mRNA levels have not been reported for X-MAID patients, it has been noted that that these patients do not exhibit compensatory upregulation of ezrin protein, and it appears that ezrin protein levels are sometimes lower than in healthy donors (11). The mechanistic basis for the coordinate downregulation of ezrin and moesin in X-MAID lymphocytes remains to be determined. Since the two genes are located on different chromosomes, cis-acting regulatory mechanisms can be ruled out. Little is known about the transcriptional control of these genes, but it seems likely that they share common transcriptional regulators that are downmodulated in response to functional defects in X-MAID T cells.

Although the processes that control expression levels of moesin^R171W^ are unknown, it is clear that protein levels drop precipitously during the transition from SP thymocyte to mature peripheral T cell. This observation points to toxicity of moesin^R171W^ and selective pressure to silence protein expression. Exactly what drives this selective pressure remains to be determined. Since expression is specifically lost in peripheral T cells (and to a lesser extent in peripheral B cells), it appears that the mutant protein interferes with one or more cellular processes that are particularly important for mature lymphocytes. We considered the possibility that loss of moesin^R171W^ is needed to allow thymic egress. However, we consistently observe a population of peripheral T cells that retain high moesin expression, so egress cannot be completely blocked. Going forward, it will be important to know if the peripheral T cells that retain moesin expression are recent thymic emigrants. A definitive answer to this question will require breeding to RAG-GFP reporter mice. In mature T cells, there are multiple processes that may be impaired by expression of the mutant protein. In addition to migration, T cell activation, proliferation or survival may be affected. With respect to activation, we found that early activation markers are upregulated normally in X-MAID SP thymocytes stimulated with TCR ligands, but we have not tested TCR sensitivity or cytokine production. Even if activation proves to be intact, the mutant protein may perturb mitosis or apoptosis, since ERM proteins are known to be involved in both processes (40–43).

Despite the extensive lymphopenia in X-MAID mice, we show that lymphocytes are key drivers of pathology in this disease. When X-MAID mice were bred onto a RagKO background, systemic inflammation was dramatically reduced. This effect was clear; whereas all X-MAID mice die by 7-10 weeks of age, X-MAID/RagKO mice survive as long as RagKO littermate controls (data not shown). It is interesting to consider how lymphocytes could drive pathology in light of the changes in moesin^R171W^ expression during T cell development. Presumably, pathology is driven by cells that express the mutant protein because MKO mice do not have overt disease (28, 44). One possibility is that recent thymic emigrants still expressing the mutant protein drive disease. Mature B cells, most of which still express significant levels of the mutant protein, may also play a role. Breeding to mice that delete T or B cells at specific points in development, and experiments in which WT Tregs are transferred into neonatal X-MAID mice will be needed to determine which populations drive disease.

One of the most interesting and important questions going forward is how the R171W mutation affects moesin’s linker activity, and how expression of this protein perturbs lymphocyte function. R171 is located within the FERM domain of the protein, in a region that makes contact with the ABD to form the autoinhibited conformation. The substitution of a bulky tryptophan residue may tend to disrupt moesin autoinhibition driving the protein toward the activated conformation. In keeping with this idea, we find that upon exposure to CCL19 or binding to integrin ligands, X-MAID thymocytes fail to undergo shape changes needed for polarized migration. The behavior of X-MAID thymocytes is reminiscent of the phenotype of B cells expressing a phospho-mimetic ezrin mutant that constitutively activates linker activity. Such cells are very round and rigid, and they fail to migrate properly both *in vitro* and *in vivo* (25, 26). Notably, defective chemotactic responses have also been reported in peripheral T cells from X-MAID patients, however the basis for these defects may differ, since moesin expression is mostly lost in these populations (11). Our work on peripheral T cells from MKO mice demonstrates the importance of characterizing the nature of migratory defects; these cells undergo normal lamellipodial-based migration in response to conventional chemokines like CCL19 and CXCL12, but exhibit defects in bleb-based motile responses to the lipid chemoattractant sphingosine-1-phosphate (29). Based on these comparisons, it seems likely that the migratory defects seen in X-MAID patients are multifaceted; thymocyte motility (and possibly also motility of bone marrow precursors) may be poisoned by overexpression of a hypermorphic mutant protein, while migration of peripheral lymphocytes may be impaired by loss of moesin expression.

Given the complex nature of moesin function in the immune cells, pathology in X-MAID patients will almost certainly prove to involve dysregulation of several important immunological processes. The murine model described here will be invaluable as we tease apart the basis of disease at the molecular, cell biological, and organismal levels.

## Data Availability Statement

The original contributions presented in the study are included in the article/**Supplementary Material**. Further inquiries can be directed to the corresponding author.

## Ethics Statement

The animal study was reviewed and approved by Institutional Animal Care and Use Committee of the Children’s Hospital of Philadelphia Research Institute.

## Author Contributions

LA conceived, performed and analyzed the experiments. TR performed and analyzed experiments involving immunoblotting and immunofluorescence microscopy. CW performed analysis of cell signaling and migration. NR performed and analyzed experiments involving live cell microscopy. SC performed and analyzed experiments involving qPCR. EP and AV assisted with analysis of bone marrow populations, with guidance and oversight from IM. JB conceived the project and oversaw its execution. LA and JB wrote the paper with critical input from all authors. All authors contributed to the article and approved the submitted version.

## Funding

This work was supported by seed funds from the University Research Foundation and the Foerderer Foundation to JB and by NIH K12GM081259 to LA. SC was supported by NIH Medical Scientist Training Program T32 GM007170. Additional support was from NIAID (R01-AI091627 to IM, F30-AI136325 to EP, F30-AI161873 to AV, T32-AI070077 to AV).

## Conflict of Interest

The authors declare that the research was conducted in the absence of any commercial or financial relationships that could be construed as a potential conflict of interest.

## Publisher’s Note

All claims expressed in this article are solely those of the authors and do not necessarily represent those of their affiliated organizations, or those of the publisher, the editors and the reviewers. Any product that may be evaluated in this article, or claim that may be made by its manufacturer, is not guaranteed or endorsed by the publisher.

## Supplementary Material

Supplementary Figure 1 Analysis of additional cell populations in X-MAID mice. 3-5 week-old WT and X-MAID male mice were sacrificed and spleen **(A)** and blood **(B)** were collected and processed for flow cytometry. Cells were gated on live single cells then CD19^+^ (B cells), NK1.1^+^ (NK cells), CD11b^+^Ly6C^+^ (Monocytes), CD11b^+^Ly6G^+^ (Neutrophils), and the proportion of cells in each population was determined. Each point represents an individual mouse. **(C)** Representative flow plot of neutrophil populations in the blood of WT or X-MAID mice. Data in A and B represent means ± StDev. Statistics were calculated using a Student’s *t* test **p < 0.01, ***p < 0.005, ****p < 0.001.

Supplementary Figure 2 CD8^+^ T cells in the spleen of X-MAID mice have an activated phenotype. 3-5 week-old WT and X-MAID male mice were sacrificed and splenocytes were analyzed by flow cytometry. CD44 and CD62L were analyzed gated on live, single, CD8^+^ cells. Data are representative of results from at least 6 individual mice.

Supplementary Figure 3 Detailed analysis of bone marrow precursor populations in WT and X-MAID mice. **(A)** Diagram of precursor populations, with markers used for analysis. **(B-F)** Bone marrow from 3-5 week-old WT or X-MAID mice was harvested, counted, and analyzed by flow cytometry after labeling with the antibody panels outlined in A. **(B)** Proportion and absolute number of LSK (%Sca1^+^cKit^+^) populations are displayed; gated on Lin^–^ population. **(C)** Proportion and absolute number of multipotent progenitor (MPP) (%CD48–CD150^–^) are displayed; gated on Lin^–^Sca1^+^cKit^+^. **(D)** Proportion and absolute number of lymphoid-primed multipotent progenitor (LMPP) (%Flt3^–^IL7Rα^+^) are displayed; gated on Lin^–^Sca1^+^cKit^+^. **(E)** Proportion and absolute number of megakaryocyte precursors (MkP) (%CD150^+^CD41^+^) are displayed; gated on Lin^–^Sca1^–^cKit^+^. **(F)** Proportion and absolute number of erythroid progenitors (EryP) (%CD105^+^CD150^+^) are displayed; gated on Lin^–^Sca1^–^cKit^+^FcγRII/III^–^CD41^–^. Note that this population is equivalent to the pre-CFU-E population (37). Data in **(B-F)** represent means ± StDev, with each point representing an individual mouse. Statistics were calculated using a Student’s *t* test **p < 0.01, ***p < 0.005, ****p < 0.001.

Supplementary Figure 4 Integrin expression is normal or slightly elevated on X-MAID thymocytes. 3 week-old WT or X-MAID male mice were sacrificed and thymocytes were labeled for flow cytometry. Cells were gated on live, single cells then CD4 SP or CD8 SP. Surface levels of CD29 (the β1 chain of VLA-4) and CD18/CD11a (LFA-1) were as high or higher than on WT cells. Data are representative of results from three individual mice.

Supplemental Movie 1 WT CD4 SP thymocytes were placed on ICAM-1 coated glass surfaces and live imaged using DIC optics every 30 seconds for 10 minutes. Cells were tracked using the manual tracking plugin on ImageJ. Color tracks follow individual cells over time.

Supplemental Movie 2 X-MAID CD4 SP thymocytes were placed on ICAM-1 coated glass surfaces and live imaged using DIC optics every 30 seconds for 10 minutes. Cells were tracked using the manual tracking plugin on ImageJ. Color tracks follow individual cells over time.
