# Supplementary figures and images for: A Murine Model of X-Linked Moesin-Associated Immunodeficiency (X-MAID) Reveals Defects in T Cell Homeostasis and Migration
Source: Front Immunol. 2022 Jan 6;12:726406. doi: 10.3389/fimmu.2021.726406 (PMC8770857; doi:10.3389/fimmu.2021.726406)

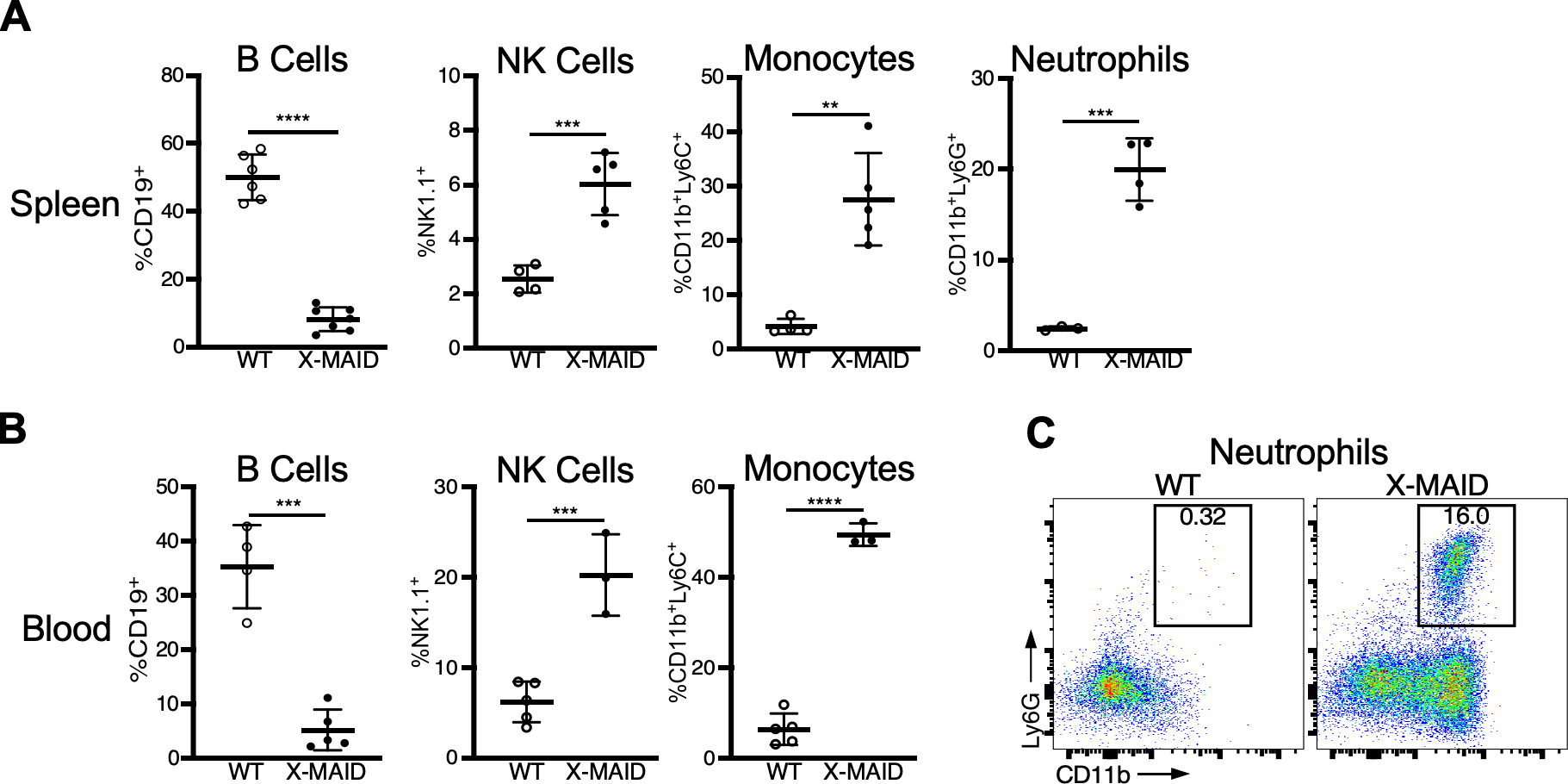

Supplement: Supplementary Figure 1 — Analysis of additional cell populations in X-MAID mice. 3-5 week-old WT and X-MAID male mice were sacrificed and spleen (A) and blood (B) were collected and processed for flow cytometry. Cells were gated on live single cells then CD19+ (B cells), NK1.1+ (NK cells), CD11b+Ly6C+ (Monocytes), CD11b+Ly6G+ (Neutrophils), and the proportion of cells in each population was determined. Each point represents an individual mouse. (C) Representative flow plot of neutrophil populations in the blood of WT or X-MAID mice. Data in A and B represent means ± StDev. Statistics were calculated using a Student’s t test **p < 0.01, ***p < 0.005, ****p < 0.001. [file Image_1.tif]

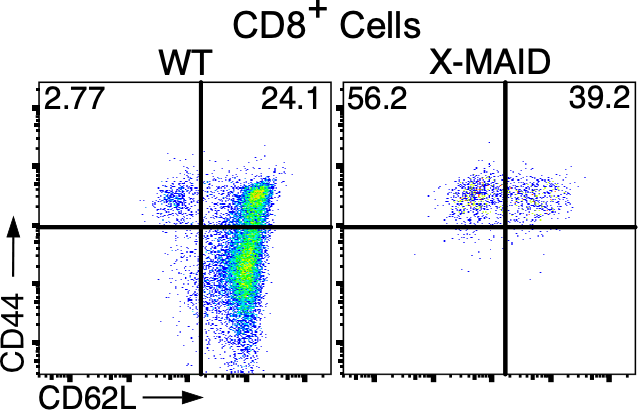

Supplement: Supplementary Figure 2 — CD8+ T cells in the spleen of X-MAID mice have an activated phenotype. 3-5 week-old WT and X-MAID male mice were sacrificed and splenocytes were analyzed by flow cytometry. CD44 and CD62L were analyzed gated on live, single, CD8+ cells. Data are representative of results from at least 6 individual mice. [file Image_2.tif]

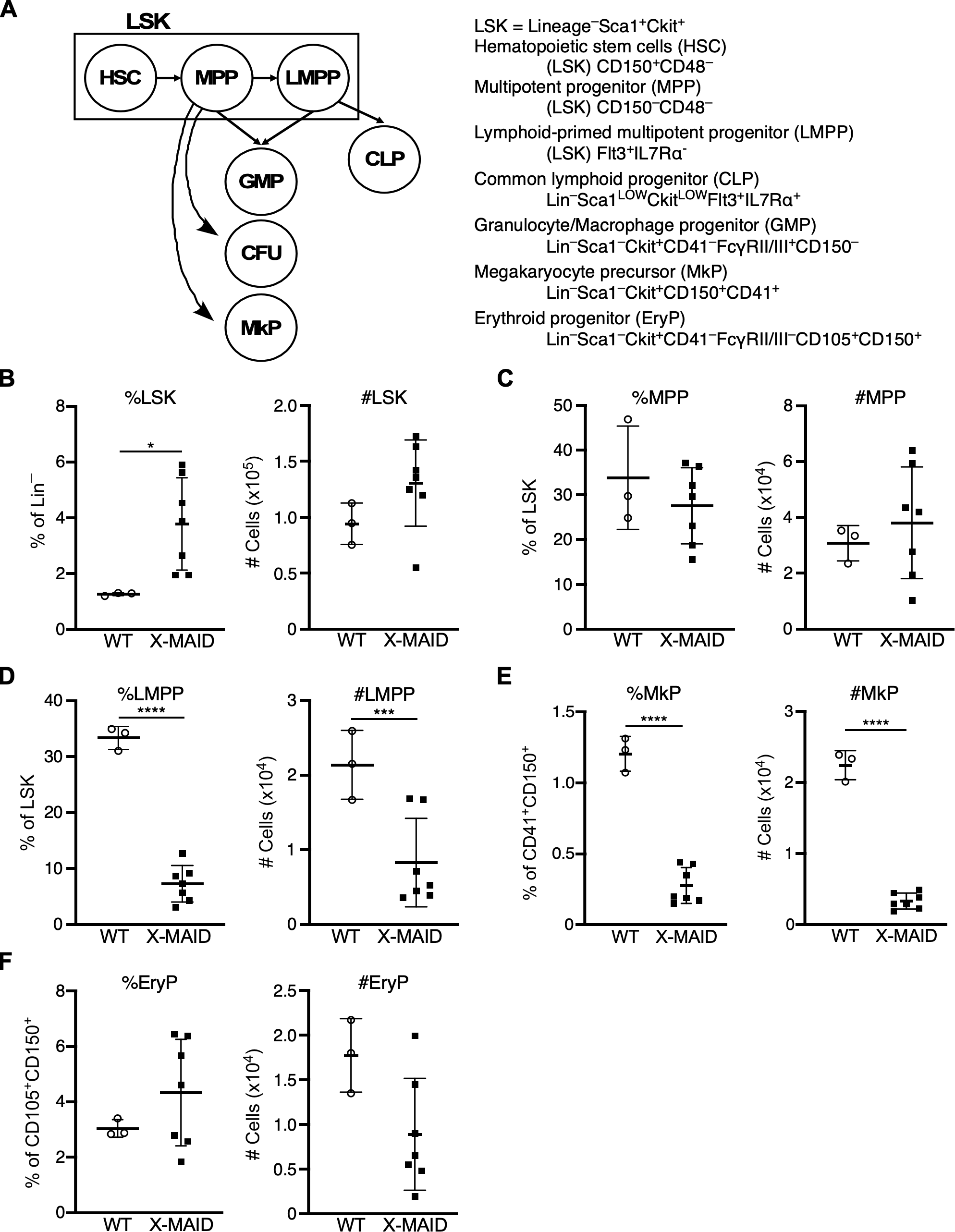

Supplement: Supplementary Figure 3 — Detailed analysis of bone marrow precursor populations in WT and X-MAID mice. (A) Diagram of precursor populations, with markers used for analysis. (B-F) Bone marrow from 3-5 week-old WT or X-MAID mice was harvested, counted, and analyzed by flow cytometry after labeling with the antibody panels outlined in A. (B) Proportion and absolute number of LSK (%Sca1+cKit+) populations are displayed; gated on Lin– population. (C) Proportion and absolute number of multipotent progenitor (MPP) (%CD48–CD150–) are displayed; gated on Lin–Sca1+cKit+. (D) Proportion and absolute number of lymphoid-primed multipotent progenitor (LMPP) (%Flt3–IL7Rα+) are displayed; gated on Lin–Sca1+cKit+. (E) Proportion and absolute number of megakaryocyte precursors (MkP) (%CD150+CD41+) are displayed; gated on Lin–Sca1–cKit+. (F) Proportion and absolute number of erythroid progenitors (EryP) (%CD105+CD150+) are displayed; gated on Lin–Sca1–cKit+FcγRII/III–CD41–. Note that this population is equivalent to the pre-CFU-E population (37). Data in (B-F) represent means ± StDev, with each point representing an individual mouse. Statistics were calculated using a Student’s t test **p < 0.01, ***p < 0.005, ****p < 0.001. [file Image_3.tif]

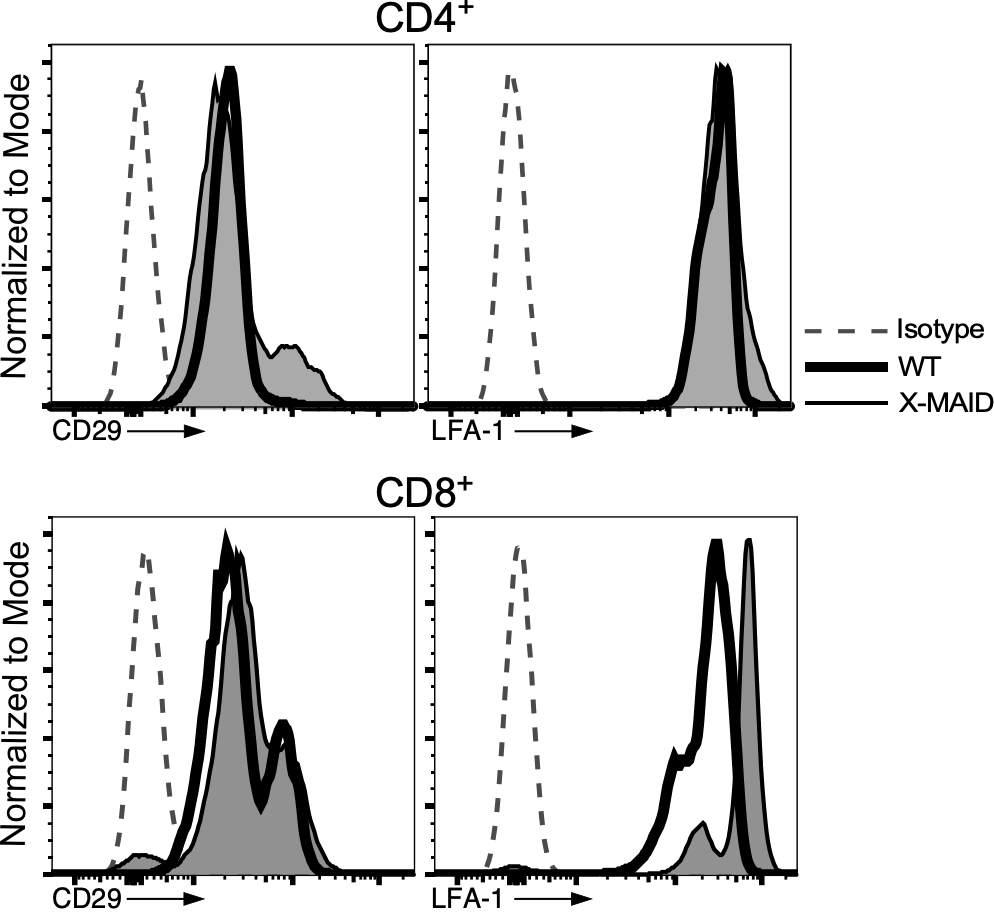

Supplement: Supplementary Figure 4 — Integrin expression is normal or slightly elevated on X-MAID thymocytes. 3 week-old WT or X-MAID male mice were sacrificed and thymocytes were labeled for flow cytometry. Cells were gated on live, single cells then CD4 SP or CD8 SP. Surface levels of CD29 (the β1 chain of VLA-4) and CD18/CD11a (LFA-1) were as high or higher than on WT cells. Data are representative of results from three individual mice. [file Image_4.tif]
